# Identification and Validation of Tumor Microenvironment-Associated Signature in Clear-Cell Renal Cell Carcinoma through Integration of DNA Methylation and Gene Expression

**DOI:** 10.3390/ijms25126792

**Published:** 2024-06-20

**Authors:** Zijian Ye, Jialiang Xu, Xin Zhang, Yifan Zhang, Deyana Ivanova, Weiyu Lu, Jianning Zhang, Fangfang Li, Xuemei Chen, Yingxiong Wang, Meijiao Wang, Biao Xie

**Affiliations:** 1 Department of Physiology, School of Basic Medical Science, Chongqing Medical University, Chongqing 400016, China2022110004@stu.cqmu.edu.cn (J.X.); 2020221770@stu.cqmu.edu.cn (X.Z.); 2020220523@stu.cqmu.cn (Y.Z.); 2019221495@stu.cqmu.edu.cn (W.L.); 2020220512@stu.cqmu.edu.cn (J.Z.); 2 Department of Biostatistics, School of Public Health, Chongqing Medical University, Chongqing 400016, China; 3 Joint International Research Laboratory of Reproduction, Development of the Ministry of Education of China, School of Public Health and Management, Chongqing Medical University, Chongqing 400016, China; lifangfang715@126.com (F.L.); chenxuemei@cqmu.edu.cn (X.C.); yxwang@cqmu.edu.cn (Y.W.); 4 Department of Medicine, Division of Endocrinology, Diabetes and Hypertension, Brigham and Women’s Hospital, Harvard Medical School, Boston, MA 02115, USA; deyana.ivanova@yahoo.com

**Keywords:** clear-cell renal cell carcinoma, molecular docking, tumor microenvironment, DNA methylation, prognostic signature

## Abstract

The tumor microenvironment (TME) is crucial in tumor development, metastasis, and response to immunotherapy. DNA methylation can regulate the TME without altering the DNA sequence. However, research on the methylation-driven TME in clear-cell renal cell carcinoma (ccRCC) is still lacking. In this study, integrated DNA methylation and RNA-seq data were used to explore methylation-driven genes (MDGs). Immune scores were calculated using the ESTIMATE, which was employed to identify TME-related genes. A new signature connected with methylation-regulated TME using univariate, multivariate Cox regression and LASSO regression analyses was developed. This signature consists of four TME-MDGs, including *AJAP1*, *HOXB9*, *MYH14*, and *SLC6A19*, which exhibit high methylation and low expression in tumors. Validation was performed using qRT-PCR which confirmed their downregulation in ccRCC clinical samples. Additionally, the signature demonstrated stable predictive performance in different subtypes of ccRCC. Risk scores are positively correlated with TMN stages, immune cell infiltration, tumor mutation burden, and adverse outcomes of immunotherapy. Interestingly, the expression of four TME-MDGs are highly correlated with the sensitivity of first-line drugs in ccRCC treatment, especially pazopanib. Molecular docking indicates a high affinity binding between the proteins and pazopanib. In summary, our study elucidates the comprehensive role of methylation-driven TME in ccRCC, aiding in identifying patients sensitive to immunotherapy and targeted therapy, and providing new therapeutic targets for ccRCC treatment.

## 1. Introduction

Renal cell carcinoma (RCC) develops from the epithelial cells of the kidney and represents over 90% of all kidney cancers. In 2020, over 179,000 people died from RCC, with a death toll exceeding 400,000 globally [1]. Clear-cell renal cell carcinoma (ccRCC) constitutes 70–75% of all RCC cases and stands as the primary cause of RCC-related deaths, rendering it the most prevalent pathological subtype of RCC [2,3]. Surgery is primarily utilized for early stage ccRCC, while immunotherapy and chemotherapy are the mainstay options for advanced and metastatic ccRCC [3]. However, the five-year survival rate for patients with advanced ccRCC is not ideal, ranging from 13% to 50% [4]. To achieve better treatment outcomes, the combined use of PD-1/PD-L1 inhibitors and multiple tyrosine kinase inhibitors has become a consensus in clinical practice. Although these treatments can improve the survival of patients, not all patients have an ideal response due to ccRCC’s resistance to chemotherapy and immunotherapy [5,6]. Thus, early identification of patients sensitive to immunotherapy and the implementation of personalized treatment plans are expected to improve the therapeutic outcomes for ccRCC patients.

Tumor microenvironments (TME) are formed by stroma, blood vessels, immune cells, and cytokines surrounding tumor cells. The mutual interactions among the components of the TME collectively determine the progression of tumors [7]. The extracellular matrix provides a structural scaffold for tumor cells, facilitating their adhesion. Simultaneously, tumor cells can secrete proteolytic enzymes to remodel the extracellular matrix, thereby increasing the invasiveness and metastatic potential [8]. CD8^+^ T cells, CD4^+^ T cells, and NK cells can eliminate tumor cells, exerting anti-tumor effects [9]. Tumor cells suppress immune cell activity by releasing inhibitory cytokines, reduce or alter antigen expression on the tumor surface to decrease the likelihood of recognition by the immune system as foreign, and recruit suppressive immune cells to induce immune tolerance, thereby evading immune recognition and clearance of tumor cells [10,11,12,13,14]. The regulation of the TME involves various mechanisms, with epigenetic modifications being a crucial part. Epigenetic modifications regulate gene transcription and expression by modulating the chromatin state, affecting gene accessibility and transcription factor binding, without altering the underlying DNA sequence [15,16]. Epigenetic changes in tumors are closely related to tumor development, among which DNA methylation is a significant part. Hypermethylation of promoter or enhancer CpG sites silences tumor suppressor genes, which in turn promotes the growth of cancer. Conversely, demethylation of promoter or enhancer CpG sites leads to activation of oncogenes, thereby promoting the development of cancer [17,18]. DNA methylation also regulates the TME. Increased methylation levels of immune-related genes lead to decreased expression. For instance, the high methylation of the promoter region of matrix metalloproteinase genes prevents transcription factors from binding, thereby reducing expression and decreasing extracellular matrix degradation. Changes in DNA methylation may also affect the function and capabilities of immune cells. The function of CD8^+^ T cells is associated with the level of methylation. Increased methylation levels reduce the effective attack of CD8^+^ T cells on tumors, thereby promoting immune evasion [19,20].

This study identified TME-related genes in ccRCC through immune scores. Additionally, information on methylation sites from the TCGA-KIRC cohort was utilized and integrated with the TME-related genes to construct a methylation regulated TME prognostic signature, validated in two external cohorts. The genes included in the prognostic signature are implicated in tumor pathogenesis and hold promise as targets for ccRCC therapy.

## 2. Results

### 2.1. The Technical Roadmap of This Study

**Figure 1:**
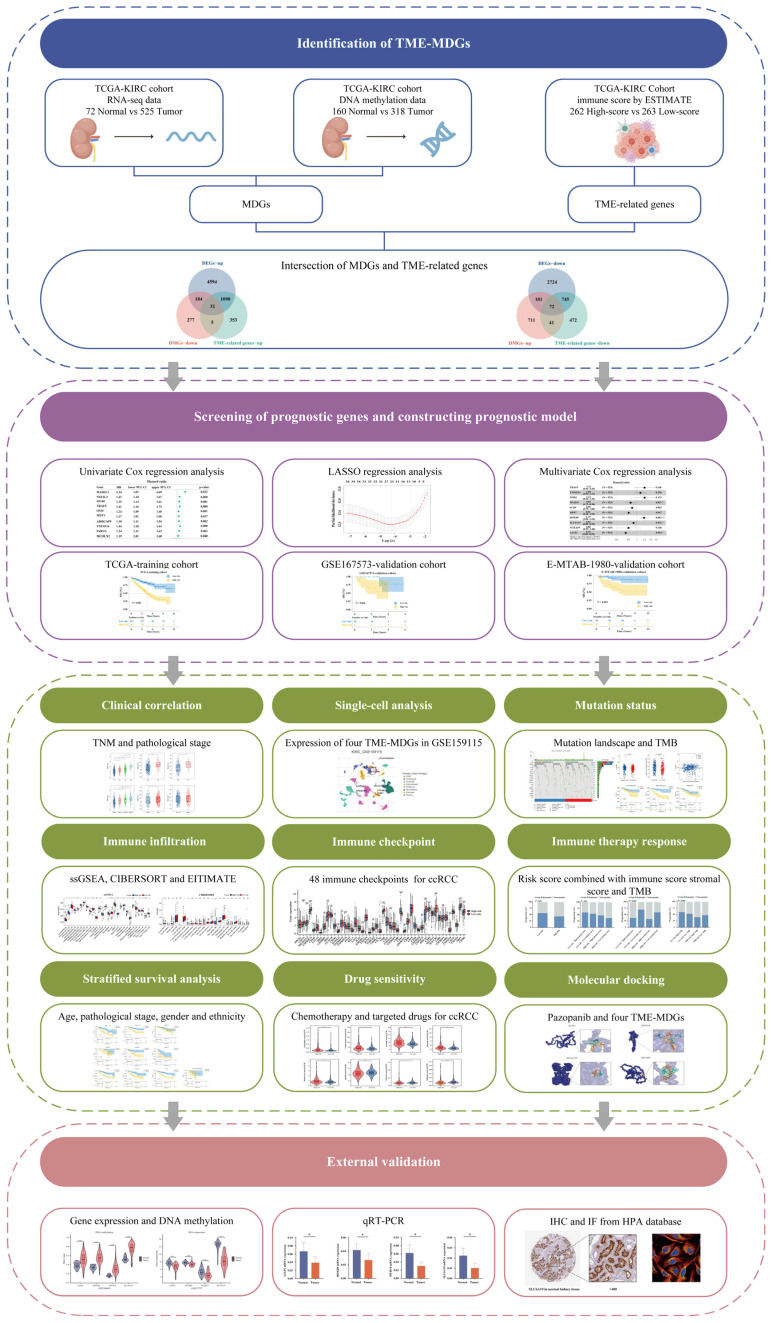
Analysis workflow for mining methylation-regulated tumor microenvironment (TME) signatures in clear-cell renal cell carcinoma (ccRCC); * *p* < 0.05, ** *p* < 0.01, *** *p* < 0.001.

Our research process is shown in Figure 1. This study is divided into four parts. The first part identifies TME-related methylation-driven genes (TME-MDGs). Differentially expressed genes (DEGs) and differentially methylated genes (DMGs) were obtained by comparing the gene expression and DNA methylation between ccRCC and normal kidney tissues. In human genomics, genes with high methylation levels typically exhibit lower expression, a phenomenon known as methylation suppression; whereas genes in a hypomethylated state often show higher expression, termed non-methylation activation or demethylation activation. MDGs are influenced by methylation suppression or demethylation activation, leading to changes in their expression levels with varying methylation levels. Therefore, candidate MDGs were identified by intersecting upregulated DEGs and downregulated DMGs, as well as downregulated DEGs and upregulated DMGs. Simultaneously, the ESTIMATE algorithm was used to identify the TME of ccRCC, dividing ccRCC samples into two groups using the median immune scores and performing a differential analysis to obtain TME-related genes. The intersection of TME-related genes and candidate MDGs yielded candidate TME-MDGs. In the second part, TME-MDGs associated with prognosis were identified. Subsequently, a prognostic signature was constructed, which was validated in two independent validation datasets. In the third part, focus was placed on the risk scores for subsequent analysis, including correlation analysis of clinical features, immune infiltration analysis, somatic mutation analysis, stratified survival analysis, immune checkpoint and drug sensitivity analysis, molecular docking, and single-cell analysis. Moreover, the expression and DNA methylation levels of four TME-MDGs were validated using the GEO dataset. Additionally, clinical ccRCC and adjacent tissue samples were collected for qRT-PCR validation. Immunohistochemistry and immunofluorescence images from the HPA database were utilized to illustrate the protein expression and subcellular localization.

### 2.2. Identifying TME-Related Genes in ccRCC

**Figure 2:**
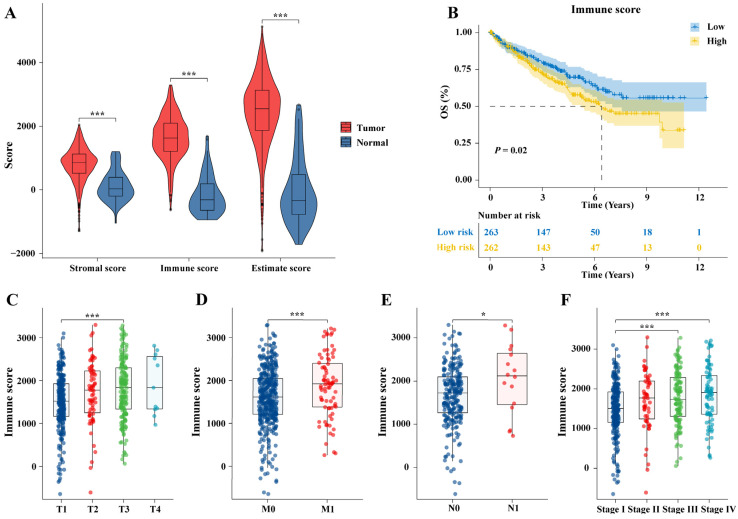
ESITMATE algorithm evaluates the TME of ccRCC. (**A**) The ESITMATE scores, immune scores, and stromal scores between ccRCC and adjacent tissue. (**B**) Kaplan–Meier (KM) survival curves of the low- and high-immune-scores groups. The immune scores are positively correlated with (**C**) T stage, (**D**) M stage, (**E**) N stage, and (**F**) pathological stage; * *p* < 0.05, *** *p* < 0.001.

**Figure 3:**
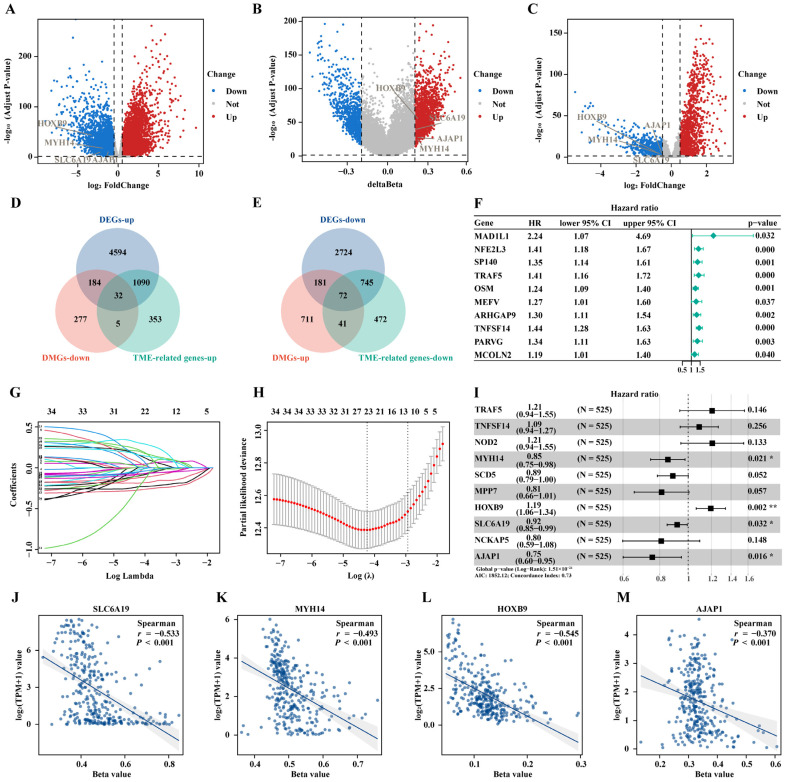
Identifying TME-methylation-driven genes (DMGs) in ccRCC and constructing a prognostic signature. Volcano plot of (**A**) differentially expressed genes (DEGs), (**B**) DMGs, and (**C**) TME-related genes. (**D**) The intersection of upregulated DEGs, downregulated DMGs, and upregulated TME-related genes, (**E**) the intersection of downregulated DEGs, upregulated DMGs, and downregulated TME-related genes. (**F**) Univariate COX regression analysis of 105 genes, listing the top 10 significant factors with *p* < 0.005. (**G**,**H**) LASSO with minimal lambda value. (**I**) Multivariable Cox regression analysis. Spearman correlation analysis between RNA expression and DNA methylation of (**J**) *SLC619*, (**K**) *MYH14*, (**L**) *HOXB9*, and (**M**) *AJAP1*; * *p* < 0.05, ** *p* < 0.01.

The immune scores, stromal scores, and ESTIMATE scores of ccRCC samples were then compared with different TMN stages and pathological stages. Compared to normal tissues, all scores are elevated in tumors (*p* < 0.001, Figure 2A). The immune scores demonstrated a statistically significant positive correlation with T (*p* < 0.001, Figure 2C), M (*p* < 0.001, Figure 2D), N (*p* < 0.05, Figure 2E), and pathological stages (*p* < 0.001, Figure 2F). The stromal scores correlated positively with M and N stages, but without statistical significance. In the T stage, an increase in immune scores was observed in the T2 and T3 stages (*p* < 0.05). No statistical difference was observed in stromal scores among pathological stage classification (Figure S1C–F). The ESTIMATE scores showed a significant increase in the T1 and T3 stages (*p* < 0.01), as well as in the M0 and M1 stages (*p* < 0.05). In terms of pathological stage, the ESTIMATE scores significantly increased in stages 1 to 3 (*p* < 0.01) and stages 1 to 4 (*p* < 0.05, Figure S1G–J). Patients were stratified into two groups based on median immune, stromal, and ESITIMATE scores. The Kaplan–Meier (KM) survival curve demonstrates that patients with low immune scores have a better overall survival (OS) (*p* = 0.02, Figure 2B). Stromal and ESITIMATE scores showed no correlation with patient prognosis (Figure S1A,B). Interestingly, as tumors progress, immune scores gradually increase, showing a trend associated with patient poor prognosis. Differential analysis was conducted using median grouping of immune scores to identify TME-related genes, revealing 1480 upregulated genes and 1380 downregulated genes (Table S1). The volcano plot depicts the expression patterns of TME-related genes (Figure 3C).

GO results indicate that TME-related genes are enriched in immune-related biological processes, such as positive regulation of cytokine production, leukocyte-mediated immunity, and activation of the immune response (Figure S2A). The KEGG results reveal that TME-related genes are associated with cytokine–cytokine receptor interaction, neuroactive ligand–receptor interaction, and the PI3K-Akt signaling pathway (Figure S2B).

### 2.3. Identification of Candidate MDGs in ccRCC

RNA-seq data were utilized to conduct difference analyses between normal kidney tissues and ccRCC to discover DEGs. A total of 9622 DEGs were obtained, with 5900 genes upregulated and 3722 genes downregulated in ccRCC (Table S2). The volcano plot depicts the expression patterns of DEGs (Figure 3A).

Differentially methylated CpGs (DMCs) were identified through a difference analysis between normal kidney tissues and ccRCC using DNA methylation data. A total of 3058 DMCs were obtained. Among them, 2217 sites showed hypermethylation, and 841 sites showed hypomethylation in ccRCC. Through the annotation of these probes, 1503 DMGs were identified, with 1005 genes characterized by hypermethylation and 498 genes characterized by hypomethylation in ccRCC (Table S3). The volcano plot depicts the expression patterns of DMGs (Figure 3B).

To identify genes in tumors that are upregulated due to low methylation and consequently lead to an increase in immune scores, genes with low methylation and upregulated expression in tumors were first intersected. Then, this set was intersected with the upregulated portion of TME-related genes, resulting in the exploration of 32 key genes (Figure 3D). To identify genes in tumors that are downregulated due to high methylation and that consequently lead to a decrease in immune scores, genes with high methylation and downregulated expression in tumors were first intersected. Then, this set was intersected with the downregulated portion of TME-related genes, resulting in 72 key genes (Figure 3E).

### 2.4. Construction and Validation of the Prognostic Prediction Signature

**Figure 4:**
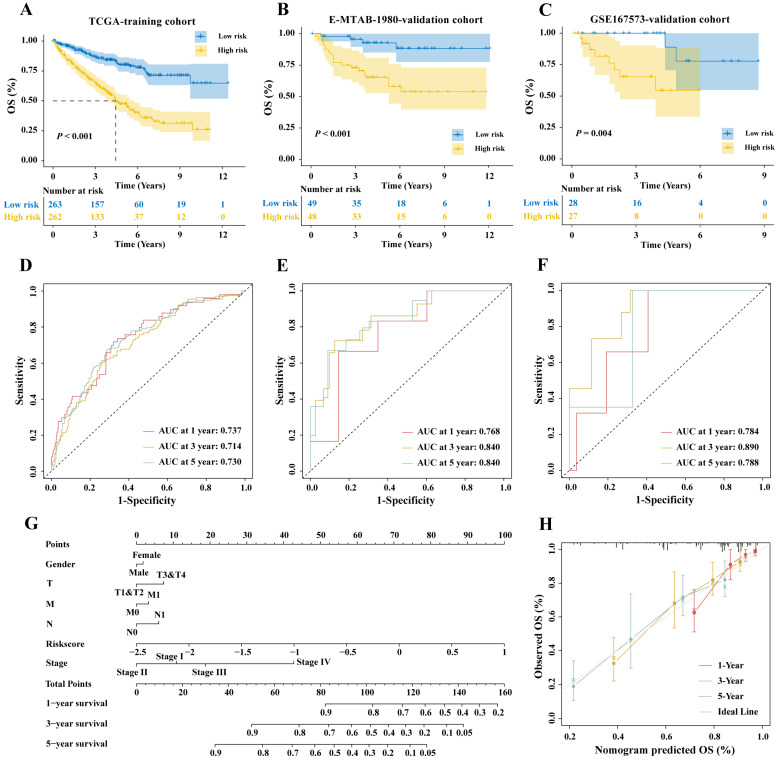
Performance of the prognostic signature. KM survival curves of patients in (**A**) the TCGA-KIRC, (**B**) E-MTAB-1980, (**C**) and GSE167573 cohorts. (**D**) Time-dependent ROC in TCGA-KIRC, (**E**) E-MTAB-1980, (**F**) and GSE167573 cohorts. (**G**) A nomogram using risk scores combined with different clinical characteristics. (**H**) Calibration curves of the nomogram.

Firstly, univariate Cox regression was conducted to analyze the correlation of 105 genes with the OS. The top ten most significant genes were selected, and a forest plot was generated (Figure 3F). Then, LASSO regression analysis was conducted to prevent overfitting (Figure 3G,H). Finally, a novel prognostic signature containing four genes was constructed using multivariate Cox regression analysis (Figure 3I). The correlation analysis between the RNA expression and the DNA methylation revealed that the expression of the four genes decreased with the increase in the methylation level, indicating that they are MDGs (Figure 3J–M). Patients were stratified by median risk scores, and the KM survival curve showed significantly lower OS in the high-risk group (*p* < 0.001, Figure 4A). In the training cohort, the AUC values of 1-year, 2-year, and 3-year predictions were 0.737, 0.714, and 0.730, respectively (Figure 4D). Additionally, a nomogram was constructed integrating the factors of TMN stage, pathological stage, gender, risk scores, and age to determine individual risk in different clinical conditions, predicting the prognosis (Figure 4G). The calibration curve reflects that the actual survival rate of ccRCC patients is consistent with the predicted survival rate from the nomogram (Figure 4H). The prognostic signature was assessed in two validation cohorts, the E-MTAB-1980 (*p* < 0.001, Figure 4B) and the GSE167573 cohorts (*p* = 0.002, Figure 4B). The KM survival curve showed similar effects in both validation sets, with AUC values of the 1-year, 2-year, and 3-year predictions being 0.768, 0.840, and 0.840, respectively, in the E-MTAB-1980 cohort (Figure 4E), and 0.784, 0.890, and 0.788, respectively, in the GSE167573 cohort (Figure 4F).

### 2.5. The Protein Expression, Cellular Localization, and Subcellular Localization of the 4 TME-MDGs

**Figure 5:**
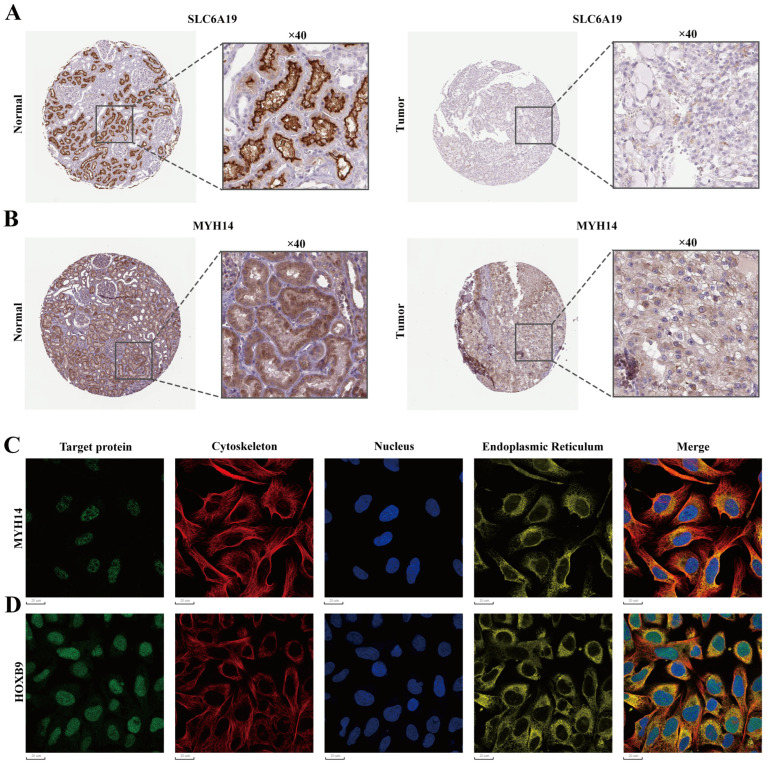
Protein expression and subcellular localization of four TME-MDGs from the HPA database. The protein expression of (**A**) SLC6A19 and (**B**) MYH14 via immunohistochemistry. The immunofluorescence images demonstrate the subcellular localization of (**C**) MYH14 and (**D**) HOXB9.

**Figure 6:**
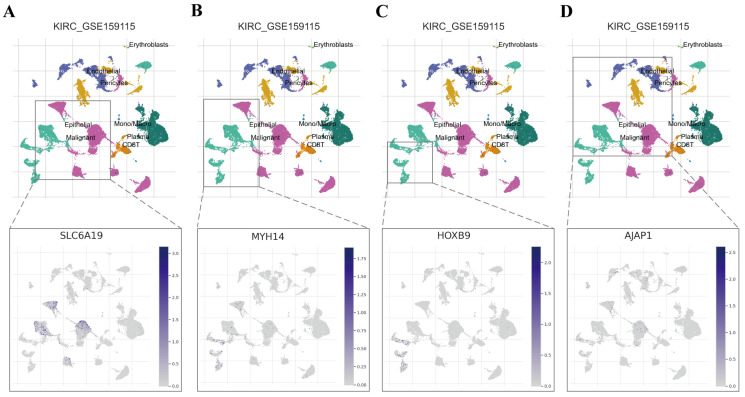
The scRNA-seq analysis of prognostic markers. Expression of (**A**) *SLC619*, (**B**) *MYH14*, (**C**) *HOXB9*, and (**D**) *AJAP1* in the single-cell dataset GSE159115.

Protein expression in normal kidney tissue and tumor tissue was further validated using the HPA database. The results were consistent with our predictions. The protein encoded by SLC6A19 is highly expressed in normal kidney tissues, and located in the proximal tubules, while it is lowly expressed in tumor tissues, mainly located in the cytoplasmic and membranous regions (Figure 5A). The protein encoded by MYH14 is highly expressed in normal kidney tissues, mainly located in the cell membrane and cytoplasm of renal tubules, whereas it is lowly expressed in tumor tissues, mainly located in the cytoplasmic and membranous regions (Figure 5B). To investigate the expression distribution of four TME-MDGs within cells, the GSE159115 dataset was analyzed on the TISCH2 website. The results show that *SLC6A19* and *MYH14* are predominantly expressed in malignant and epithelial cells (Figure 6A,B), while *HOXB9* is only expressed in malignant cells (Figure 6C). *AJAP1* is expressed in malignant, epithelial, and endothelial cells, but at a low level (Figure 6D). It is worth noting that all four TME-MDGs are expressed in malignant cells, further validating the accuracy of our TME-MDGs selection and their potential roles in regulating the TME, thereby influencing tumor progression. Additionally, patients were grouped based on the median expression level of each TME-MDG of prognostic signature, and KM survival curves were plotted. The results show that high expression of *AJAP1*, *SLC6A19*, and *MYH14* is beneficial for prognosis, while high expression of *HOXB9* is detrimental to prognosis (Figure S3A–D).

### 2.6. Analysis of Immune Cell Infiltration

**Figure 7:**
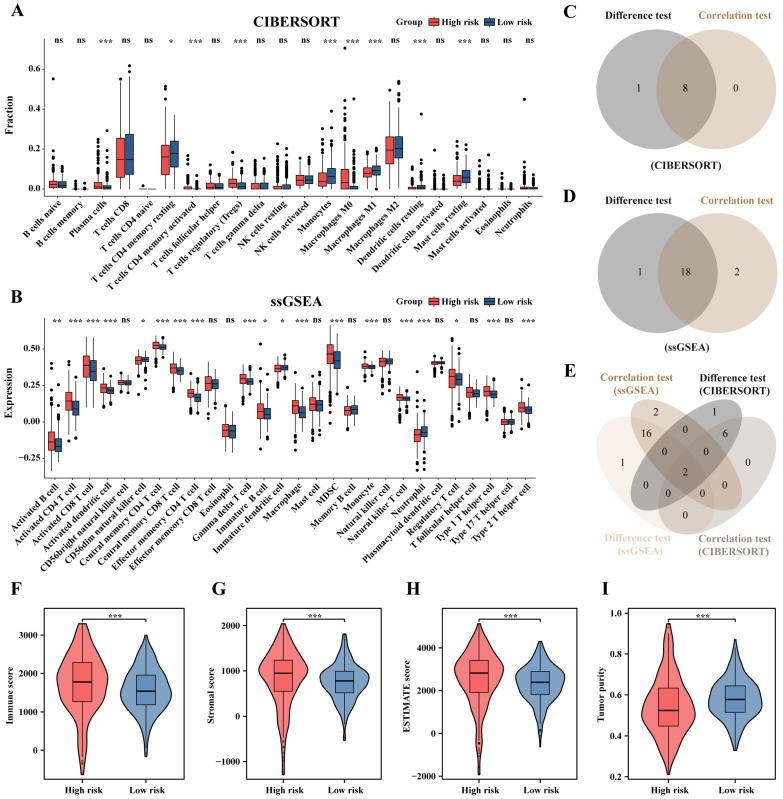
Immune cell infiltration based on prognostic signature. (**A**) CIBERSORT and (**B**) ssGSEA. The Venn diagram shows the intersection of immune cells with *p* < 0.05 in the difference and correlation analysis in (**C**) CIBERSORT, (**D**) ssGSEA, (**E**) and both. Comparison of (**F**) immune scores, (**G**) stromal scores, (**H**) ESTIMAT scores, and (**I**) tumor purity between high- and low-risk groups; * *p* < 0.05, ** *p* < 0.01, *** *p* < 0.001, ns, not significant.

CIBERSORT showed a higher proportion of activated immune cells, including plasma cells and activated T cells CD4^+^ memory, in the high-risk group, while the low-risk group showed a higher proportion of resting immune cells, including resting T cells CD4^+^ memory, resting Dendritic cells, and resting Mast cells (Figure 7A). Correlation analysis between risk scores and immune cell proportions revealed nine immune cell types correlated with risk scores (Figure S5). ssGSEA showed that 19 immune cell-related features exhibited statistical differences between the two groups, with 16 features higher in the high-risk group and 3 features higher in the low-risk group (Figure 7B). Consistent with the results from CIBERSORT, activated immune cells are significantly elevated in the high-risk group. Correlation analysis between risk scores and immune cell-related features revealed 18 immune cell types correlated with risk scores (Figure S4). Venn diagrams displayed statistically significant immune cells or immune cell-related features in differential and correlation analyses of both CIBERSORT and ssGSEA (Figure 7C,D). Notably, Monocytes and Tregs were statistically significant in both CIBERSORT and ssGSEA analyses (Figure 7E). Additionally, in ESTIMATE analysis, the high-risk group showed higher immune scores, stromal scores, and ESTIMATE scores, with lower tumor purity (*p* < 0.001, Figure 7F–I)

### 2.7. Prediction of Immunotherapy, Chemotherapy, and Targeted Therapy

**Figure 8:**
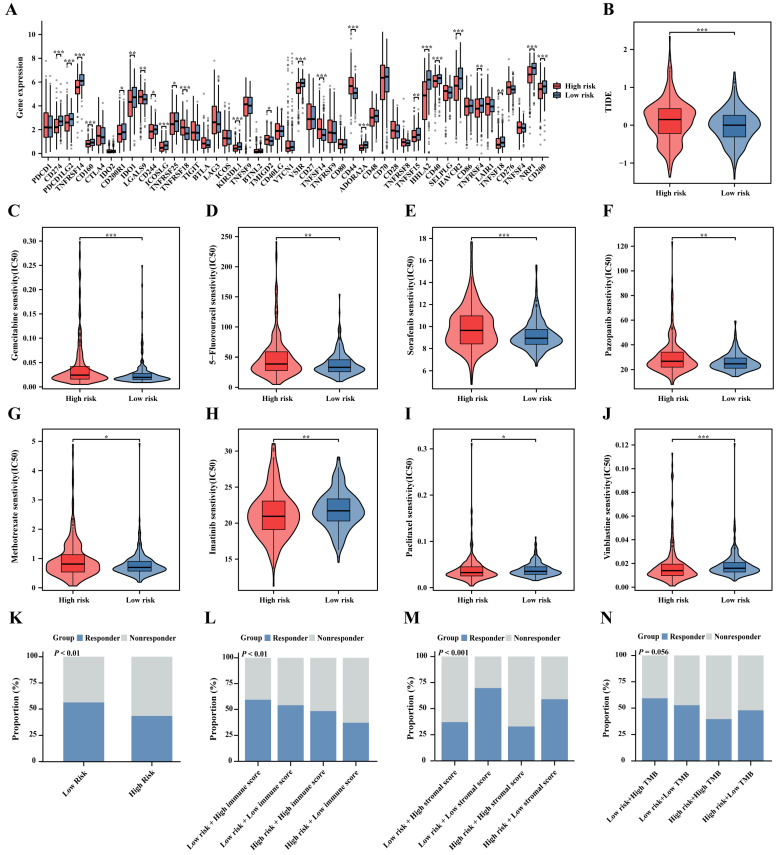
Assessment of ccRCC treatment based on prognostic signature. (**A**) Differential expression of immune checkpoints, (**B**) tumor immune dysfunction and exclusion (TIDE) scores between high- and low-risk groups, and (**C**–**J**) violin plots of the sensitivity values of eight anticancer drugs. (**K**) Risk scores, (**L**) combined risk scores and immune scores, (**M**) combined risk scores and stromal scores, and (**N**) combined risk scores and TMB prediction for patient immune therapy response ratio; * *p* < 0.05, ** *p* < 0.01, *** *p* < 0.001.

Immune checkpoint blockade (ICB) is now widely used in tumor immunotherapy. Our results indicate that most immune checkpoint levels are upregulated in the low-risk group (Figure 8A), suggesting greater benefits from ICB therapy in this group. We further quantified the tumor response to immunotherapy using Tumor Immune Dysfunction and Exclusion (TIDE) scores. The TIDE scores were lower in the low-risk group (*p* < 0.001, Figure 8B), suggesting potentially better response to ICB therapy (*p* < 0.01, Figure 8K). Interestingly, when the risk scores are combined with the immune scores, the proportion of patient immune responses gradually decreases in the low-risk group high-immune-scores subset, low-risk group low-immune-scores subset, high-risk group high-immune-scores subset, and high-risk group low-immune-scores subset (*p* < 0.01, Figure 8L). It indicates that the combined risk scores and immune scores are reliable indicators for predicting patient immune therapy response. More notably, when the risk scores are combined with the stromal scores, the proportion of immune therapy response in the high-stromal-scores subset of the low-risk group is significantly reduced compared to the low–stromal-scores subset of the low-risk group, and, similarly, the proportion of immune therapy response in the high-stromal-scores subset of the high-risk group is significantly reduced compared to the low-stromal-scores subset of the high-risk group (*p* < 0.001, Figure 8M). Therefore, the combination of risk and stromal scores are not only reliable predictors of patient response to immunotherapy, but they are more predictive than the combination of risk and immune scores. Similarly, when the risk scores are combined with tumor mutation burden (TMB), a similar trend towards improving the prediction of patient immune therapy response was observed, but it did not reach statistical significance (Figure 8N). Additionally, to explore effective drugs for ccRCC, we collected 16 chemotherapy and targeted drugs (Sunitinib, Sorafenib, pazopanib, Cabozantinib, Axitinib, Tivozanib, Temsirolimus, Docetaxel, Paclitaxel, Vinblastine, Vorinostat, Gemcitabine, Cisplatin, Imatinib, 5-Fluorouracil, and Methotrexate). In addition, Gemcitabine, 5-Fluorouracil, Sorafenib, pazopanib, and Methotrexate have higher inhibitory concentrations in the high-risk group, while Imatinib, Paclitaxel, and Vinblastine have higher inhibitory concentrations in the low-risk group (Figure 8C–J).

### 2.8. The Drug Sensitivity and Molecular Docking Analysis of Four TME-MDGs

**Figure 9:**
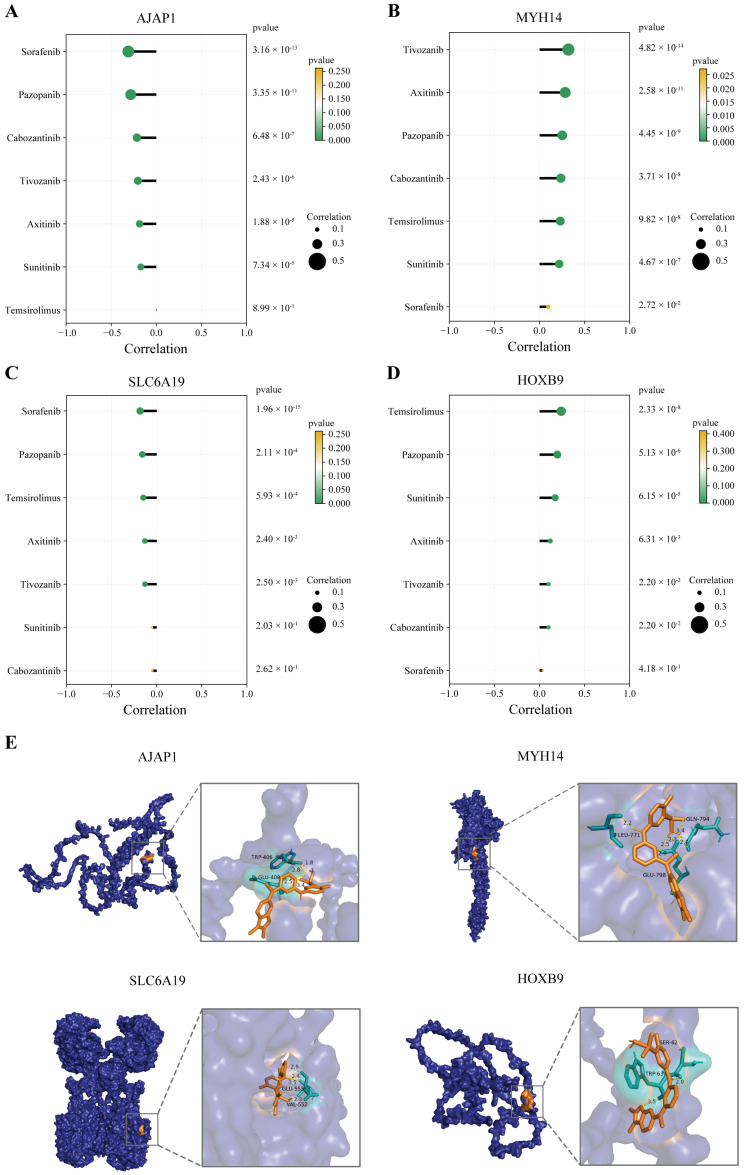
The drug sensitivity and molecular docking analysis of four TME-MDGs. (**A**–**D**) The lollipop chart displays the correlation coefficients and P-values between the expression of four TME-MDGs and first-line therapeutic drugs for ccRCC. (**E**) Prediction of the binding site between prognostic markers and pazopanib, where deep blue represents the protein, orange represents pazopanib, cyan represents the protein docking residues, and yellow dashed lines represent potential hydrogen bonds.

To investigate the relationship between the four TME-MDGs and first-line therapies for ccRCC, correlation analyses were conducted between the four TME-MDGs and seven first-line drugs used to treat ccRCC (Sorafenib, pazopanib, Cabozantinib, Tivozanib, Axitinib, Sunitinib, and Temsirolimus). *AJAP1* and *SLC6A19* were negatively correlated with the sensitivity of these seven drugs (Figure 9A,C), while *MYH14* and *HOXB9* showed positive correlations. Particularly noteworthy was pazopanib, as it exhibited significant correlations with all four TME-MDGs in the correlation analysis (*p* < 0.001, Figure 9A–D), and its half-maximal inhibitory concentration (IC50) was higher in the high-risk group (*p* < 0.01, Figure 8F).

To explore the binding sites between the four TME-MDGs and pazopanib, molecular docking analyses were conducted. The results showed that pazopanib forms four hydrogen bonds with AJAP1 (TRP-406 and GLU-408), seven hydrogen bonds with MYH14 (LEU-771 and GLN-794 and GLU-798), four hydrogen bonds with SLC6A19 (GLU-553 and VAL-552), and two hydrogen bonds with HOXB9 (SER-62 and TRP-63) (Figure 9E). Importantly, the docking energies between pazopanib and the four TME-MDGs were all less than −5 kcal/mol, indicating favorable binding interactions (Table 1).

### 2.9. Mutational Landscape in ccRCC

**Figure 10:**
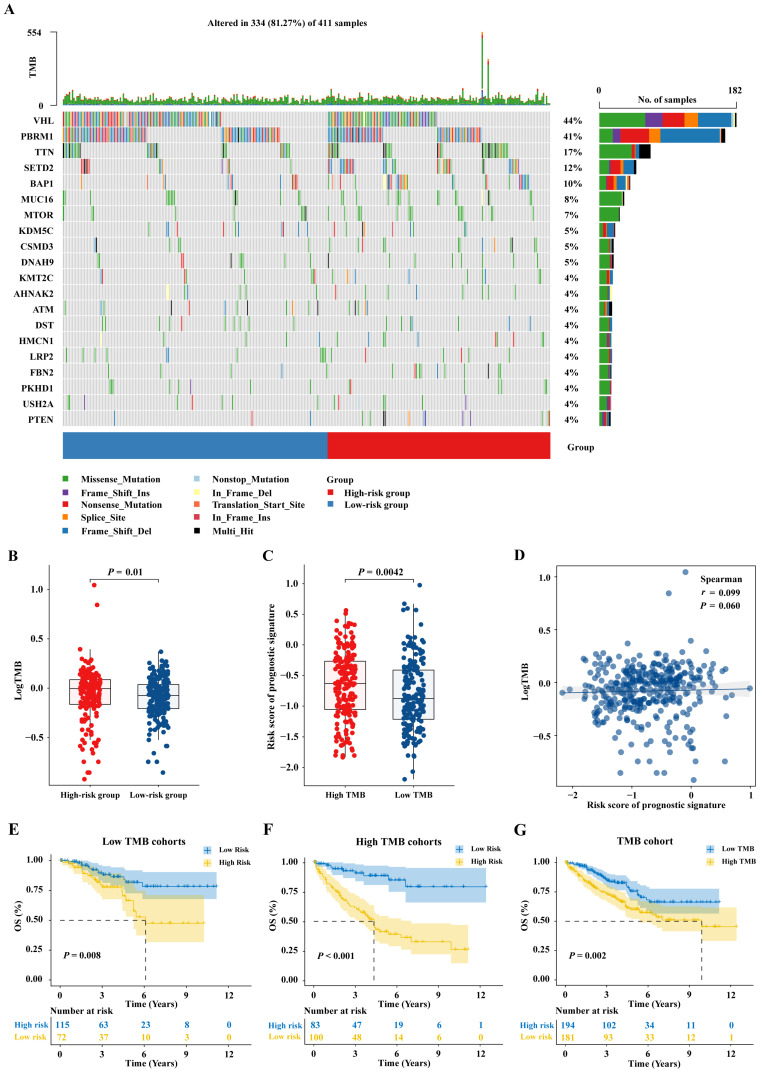
Mutational landscape in ccRCC. (**A**) The tumor mutation waterfall plot of ccRCC. (**B**,**C**) Relationship between the risk scores and tumor mutation burden (TMB). (**D**) Spearman correlation analysis between risk scores and TMB. (**E**,**F**) The KM survival curves for both low-TMB and high-TMB subsets. (**G**) The KM survival curves of ccRCC patients in the low- and high-TMB groups.

The somatic mutation data were processed and plotted as a waterfall plot. It was observed that the mutation frequencies of VHL, PBRM1, and TTN, the top 3 genes with the highest mutation frequencies, were decreased in the high-risk group (Figure 10A). Additionally, by calculating the TMB for each sample, it was found lower TMB was present in low-risk patients (*p* < 0.05, Figure 10B), and the low-TMB patients exhibited lower risk scores (*p* < 0.01, Figure 10C). However, Spearman correlation analysis showed a weak positive correlation between risk scores and TMB, with *p*-values not reaching statistical significance (Figure 10D). The relationship between TMB and patient prognosis was further investigated. Patients with low TMB experienced better OS (*p* < 0.01, Figure 10G). Within the low-TMB subgroup, low-risk patients exhibit better prognosis (*p* < 0.01, Figure 10E). The results in the high-TMB subgroup are similar to the low-TMB subgroup, but the discriminatory ability of KM curves was higher in the high-TMB subgroup. This may suggest that the prognostic signature has higher accuracy in the high-TMB subgroup (*p* < 0.001, Figure 10F).

### 2.10. Gene Alteration Analysis

**Figure 11:**
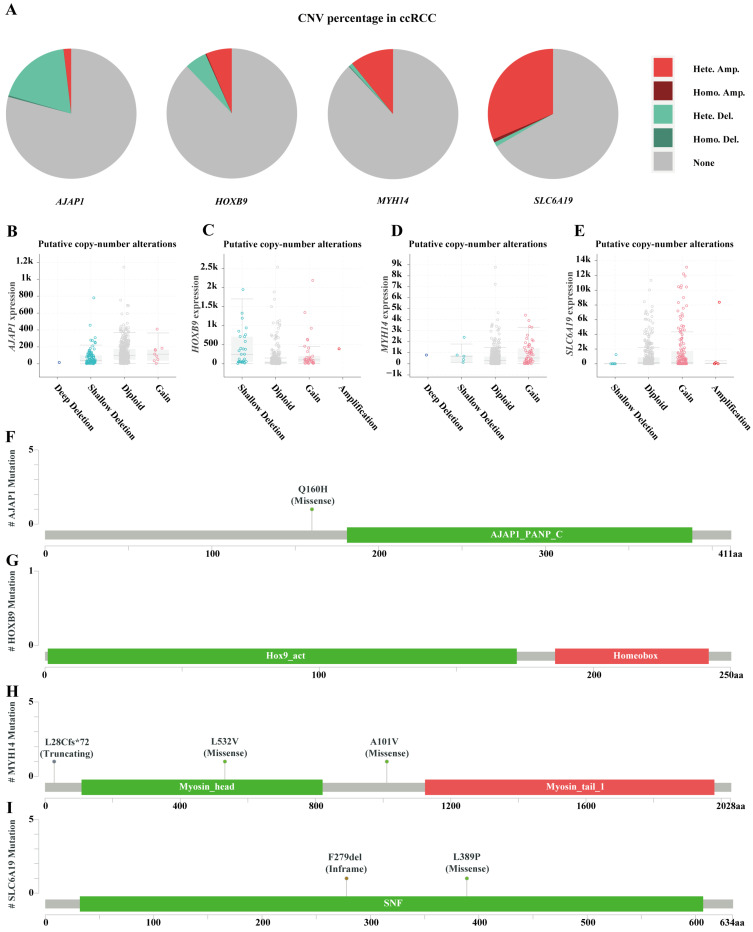
Genomic alterations of the four TME-MDGs. (**A**) Evaluation of copy number variation changes in *AJAP1*, *HOXB9*, *MYH14*, and *SLC6A19*. Comparison of (**B**) *AJAP1*, (**C**) *HOXB9*, (**D**) *MYH14*, and (**E**) *SLC6A19* expression levels across different types of copy number variations. Diagram showing the mutation types, quantities, and sites for (**F**) *AJAP1*, (**G**) *HOXB9*, (**H**) *MYH14*, and (**I**) *SLC6A19*.

Firstly, an analysis was conducted on the distribution of copy number variation (CNV) types for the four TME-MDGs in ccRCC. Predominantly, heterozygous amplifications were observed in HOXB9, *MYH14*, and *SLC6A19*, likely resulting in an increase in copy number. In contrast, heterozygous deletions were mainly observed in *AJAP1*, potentially leading to a reduction in copy number (Figure 11A). Subsequently, an analysis was performed on gene expression corresponding to different CNV types. It was observed that the expression of *AJAP1* in the gain state showed no significant change compared to the diploid and normal states with no CNV (Figure 11B). Interestingly, it was found that the shallow deletion state of HOXB9 promoted gene expression (Figure 11C). Finally, an examination was carried out on the mutation types, locations, and quantities for the four TME-MDGs. The results revealed a missense mutation in *AJAP1* at the Q160H site (Figure 11F), missense mutations in MYH14 at the L28Cfs*72, L532V, and A101V sites (Figure 11H), an in-frame variation in *SLC6A19* at the F279del site, and a missense mutation at the L389P site (Figure 11I). No mutations were observed in *HOXB9* (Figure 11G).

### 2.11. Risk Scores Combined with Clinical Features

**Figure 12:**
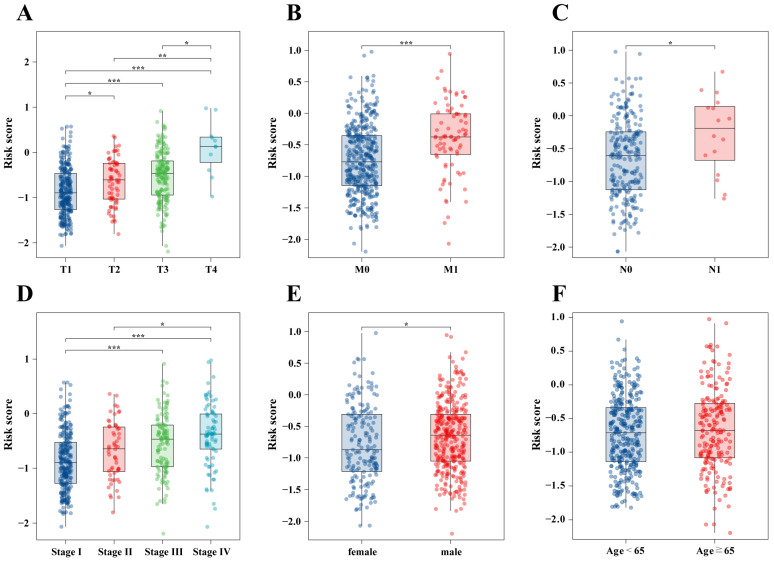
Differences between prognostic signature and pathological stage. (**A**) T stage, (**B**) M stage, (**C**) N stage, (**D**) pathological stage, (**E**) gender, and (**F**) age; * *p* < 0.05, ** *p* < 0.01, *** *p* < 0.001.

Various clinical factors affecting the risk scores were analyzed, revealing an increase in risk scores with the primary tumor size as well as the pathological stage (Figure 12A,D). Patients with distant metastasis showed significantly higher risk scores compared to those without distant metastasis (*p* < 0.001, Figure 12B), while patients with lymph node involvement demonstrated markedly higher risk scores than those without (*p* < 0.05, Figure 12C). The risk scores are significantly higher in males (*p* < 0.05, Figure 12E). There was no statistically significant difference in risk scores between patients aged ≥ 65 and those aged < 65 (Figure 12F).

### 2.12. Stratified Survival Analysis

**Figure 13:**
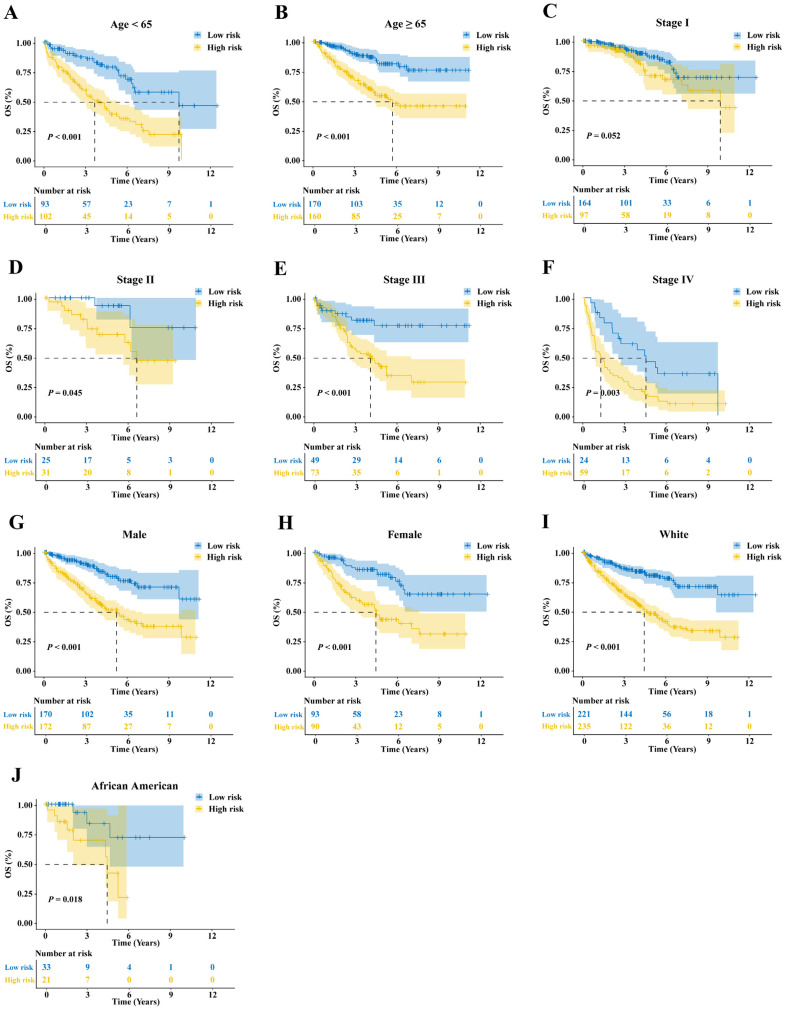
Stratified survival analysis. KM survival curves predict the OS of the TCGA-KIRC cohort in (**A**) Age < 65 years, (**B**) Age ≥ 65 years, (**C**) Stage 1, (**D**) Stage 2, (**E**) Stage 3, (**F**) Stage 4, (**G**) Male, (**H**) Female, (**I**) White, and (**J**) African American.

Considering that clinical factors may influence patient prognosis, ccRCC subtypes were stratified based on age (age < 65 and age ≥ 65) (Figure 13A,B), pathological stage (Figure 13C–F), gender (male and female) (Figure 13G,H), and ethnicity (White and African American) (Figure 13I,J). Utilizing the prognostic signature, we categorized samples from each subtype into high- and low-risk groups. By comparing the OS of the two groups within specific subtypes, the stability of the prognostic signature for each clinical subtype of ccRCC was evaluated. Survival curves revealed that all subgroups exhibited better OS in the low-risk group, indicating the applicability of the prognostic signature constructed in this study across various clinical subtypes of ccRCC.

### 2.13. External Datasets and qRT-PCR Validation

**Figure 14:**
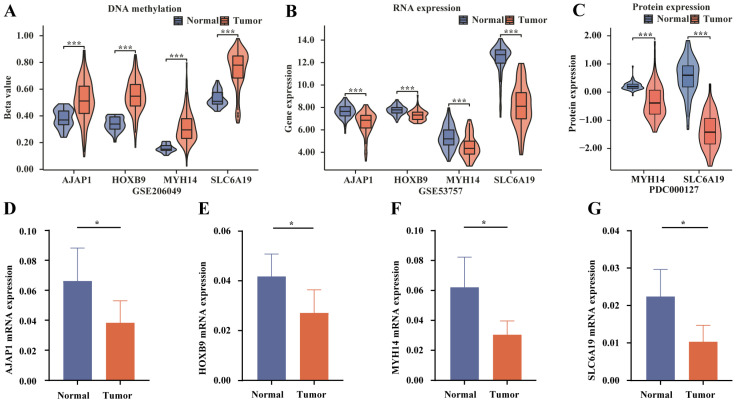
DNA methylation, gene expression, and protein expression of four TME-MDGs and qRT-PCR validation. Differences in DNA methylation, RNA expression, and protein expression between ccRCC and adjacent tissues in (**A**) GSE206049, (**B**) GSE53757, and (**C**) PDC000127 datasets. qRT-PCR validation of (**D**) *AJAP1*, (**E**) *HOXB9*, (**F**) *MYH14*, and (**G**) *SLC6A19* in ccRCC and adjacent tissues; * *p* < 0.05, *** *p* < 0.001.

To validate the results of high methylation and low expression of four TME-MDGs in tumors that were observed in the TCGA database, the GSE20649 dataset was examined to verify the DNA methylation levels of the four TME-MDGs. The results showed elevated DNA methylation levels of the four TME-MDGs in tumors (*p* < 0.001, Figure 14A). Additionally, the GSE53757 dataset was used to validate the gene expression of the four TME-MDGs, which indicated decreased gene expression of all four TME-MDGs in tumors (*p* < 0.001, Figure 14B). PDC000127 was used to validate the protein expression levels of MYH14 and SLC6A19. The results showed that their protein levels were decreased in tumor tissues (*p* < 0.001, Figure 14C). Additionally, the expression levels of MYH14 and SLC6A19 proteins in the context of alterations in signaling pathways were also observed. The results revealed that under conditions of alterations in the mTOR pathway, WNT pathway, p53/Rb pathway, RTK pathway, MYC/MYCN alterations, chromatin modification changes, NRF2 pathway alterations, SWI-SNF complex alterations, and HIPPO pathway alterations, both MYH14 (Figure S6) and SLC6A19 (Figure S7) exhibited decreased expression levels. Furthermore, we further validated the bioinformatics analysis results by collecting ccRCC and adjacent normal samples for qRT-PCR validation. Results showed that the four TME-MDGs, *AJAP1* (Figure 13D), *HOXB9* (Figure 14E), *MYH14* (Figure 14F), and *SLC6A19* (Figure 14G), were downregulated in ccRCC tissues (*p* < 0.01), which is consistent with bioinformatics analyses.

## 3. Discussion

With advances in sequencing technologies, research on the pathogenesis of tumors has become richer and more in-depth. At the same time, many effective biomarkers and therapeutic targets have been identified for clinical practice. Currently, it is verified that VHL, HIF-1α, and EGFR can predict ccRCC prognosis and how these patients will react to targeted therapy [21,22,23,24]. However, effective biomarkers remain deficient. TME participates in tumor growth and progression, immune evasion, and drug resistance. Biomarkers related to TME have shown promising abilities to predict patient prognosis [25]. The regulation of TME involves multiple mechanisms, where epigenetic modifications play a crucial role, particularly DNA methylation [26,27]. Investigating the modulation of TME using methylation and applying it to assess the prognosis, immune therapy, chemotherapy, and targeted drug response in ccRCC patients is urgently needed. This study combined DNA methylation and RNA-seq data to pinpoint MDGs. Using ESTIMATE to characterize the TME of ccRCC patients, patients were stratified based on median immune scores for differential analysis to identify TME-related genes. By intersecting TME-related genes with MDGs and applying univariate, multivariate Cox regression, and LASSO regression analyses, a new predictive signature comprising four TME-MDGs was developed.

The TME-MDGs, including *HOXB9*, *MYH14*, *SLC6A19*, *and AJAP1*, are all associated with the formation of the TME of ccRCC. *HOXB9* can enhance tumor invasion and metastasis by regulating the expression of genes involved in epithelial–mesenchymal transition. The overexpression of *HOXB9* plays a dual role in various cancers. Specifically, its overexpression induces resistance to chemotherapy drugs in pancreatic and ovarian cancers. On the other hand, the overexpression of *HOXB9* also serves as a beneficial prognostic factor in certain cancers, including gastric cancers. It was observed that the main types of CNVs in *HOXB9* were heterozygous amplification and heterozygous deletion, with the proportions of both being similar. Considering that heterozygous amplification and heterozygous deletion have opposite effects on gene expression, this may imply that CNVs are not the primary factors influencing *HOXB9* expression. Interestingly, compared to diploid *HOXB9* expression, both gain and shallow deletion increased *HOXB9* expression. This may be related to transcriptional compensation, complex gene regulatory networks, and single-copy gene effects. However, the specific mechanism requires further investigation. Truncating mutations in *PBRM1* in ccRCC may lead to transcriptional upregulation of *HOXB9* due to elevated DNA methylation levels, thereby reducing gene expression [28]. In accordance with this study, it was observed that *HOXB9* is regulated by DNA methylation, and a poor prognosis in ccRCC is associated with high expression of *HOXB9*.

*MYH14* encodes a protein related to muscle contraction, potentially impacting tumor cell migration and invasion by regulating cytoskeletal remodeling and movement during the migration process. Surcel A et al. [29] indicates that 4-hydroxyacetophenone increases the expression of *MYH14*, which induces changes in the morphology of pancreatic cancer cells, thereby inhibiting their migration and invasion capabilities. Meanwhile, *MYH14* localizes primarily to the apical membrane of intercalated cells in the mouse collecting duct and may regulate membrane and organelle trafficking in the kidney [30]. Consistent with the above study, the adverse outcome for ccRCC patients is related to low expression of *MYH14*. We speculate that *MYH14* may affect cell migration and invasion by regulating the transport of renal membranes and organelles, influencing the remodeling of the cytoskeleton. However, this hypothesis requires further experimental research in the future. The primary type of CNVs for *MYH14* in ccRCC is heterozygous amplification, which theoretically would lead to increased gene expression. However, its expression is decreased compared to normal kidney tissue. We believe that the high DNA methylation of *MYH14*, affecting the transcription or translation process, is a potential reason for this discrepancy. Additionally, our study is the first to propose that the expression of *MYH14* is epigenetically regulated in ccRCC, providing a basis for further elucidating the mechanism of action of *MYH14*.

*SLC6A19* encodes a protein known as an amino acid transporter, which is essential in the kidneys and intestines and is primarily responsible for the absorption and reabsorption of neutral amino acids. The expression of *SLC6A19* decreases gradually in different stages (I to IV) of ccRCC. This decreasing trend in expression correlates with the severity and progression stages of the disease [31]. The decrease in *SLC6A19* expression may reflect the response of renal epithelial cells to injury, affecting the renal drug metabolism and transport functions [32]. Similar results were found in our study, where the poor outcome of ccRCC is associated with low expression of *SLC6A19*. It is speculated that the progressive deterioration of ccRCC affects the transport of neutral amino acids, leading to an unfavorable outcome for patients. In the CNVs analysis, similar to *MYH14*, the primary type of CNVs for *SLC6A19* is heterozygous amplification. However, its gene expression is reduced. We speculate that the elevated DNA methylation levels in the tumor might be the reason for this decrease in expression. Additionally, for the first time, it has been elucidated that the *SLC6A19* expression in ccRCC is epigenetically regulated, providing a foundation for further research into its function in ccRCC.

*AJAP1* is crucial in regulating cell adhesion and migration by actively contributing to the establishment and preservation of cell–cell junctions, thus potentially impacting cell migration by modulating the adhesion between neighboring cells. Diminished expression of *AJAP1* serves as an unfavorable prognostic indicator in hepatocellular carcinoma, glioma, and esophageal cancer [33,34,35]. Additionally, previous studies have found *AJAP1* to be highly methylated in glioblastoma, neuroglioma, prostate cancer, and endometrial cancer. Consistent with previous research, elevated methylation levels of *AJAP1* were also found in ccRCC, indicating its role as an MDG. Additionally, the primary type of CNVs for *AJAP1* is heterozygous deletion, which reduces gene expression. The low expression of *AJAP1* in ccRCC is likely due to the synergistic effect of high DNA methylation and heterozygous deletion. Reduced *AJAP1* expression is related to an adverse prognosis of ccRCC. We speculate that this may be due to the reduced adhesive capability between tumor cells, thereby promoting tumor cell migration.

It is noteworthy that, for the first time, the potential regulatory relationship between MYH14 and SLC6A19 in the context of epigenetic regulation has been elucidated, considering DNA methylation, RNA expression, and protein expression. Interestingly, a decrease in the protein expression of MYH14 and SLC6A19 was observed under conditions of alterations in multiple signaling pathways. L. Francesco et al. have elucidated how tumor stem cells influence cell fate through signaling pathways such as Notch, Wnt, and Hedgehog [36]. In this study, a significant decrease in the protein expression of MYH14 and SLC6A19 was observed under conditions of WNT pathway alterations. It is speculated that MYH14 and SLC6A19 may be involved in the dynamic development of tumor stem cells through epigenetic mechanisms, as observed in our study. Considering the findings that MYH14 and SLC6A19 can impact TME progression through epigenetic regulation, targeting these signaling pathways may offer a dual strategy to disrupt the dynamics of tumor stem cells and epigenetic regulation within the TME, thereby delaying tumor progression. This provides hope for a more effective therapeutic approach.

Most studies on prognostic markers for ccRCC concentrate on a single biomarker or omics level. However, tumorigenesis is a multifactorial, multistage complex process, and studying prognostic markers for tumors from a multi-omics perspective can enhance their prognostic value [37]. Moreover, integrating multiple biomarkers into an aggregated model can improve prediction accuracy compared to single biomarkers alone [38]. He et al. [39] also developed a six-gene prognostic signature of TME using the ESTIMATE algorithm, with AUC values of 0.718 in the training set and 0.705 in the validation set. In comparison, our prognostic signature, developed by integrating gene expression and DNA methylation levels, only includes four genes but exhibits better predictive accuracy. The AUC values in the training set range from 0.714 to 0.737, while in the validation sets E-MATE-190 and GSE1677573, they range from 0.768 to 0.840 and from 0.784 to 0.890, respectively. Our integrated gene expression and DNA methylation-based prognostic signature demonstrates superior efficacy in predicting patient OS and has been validated in larger, more diverse sample cohorts. In contrast to previous prognostic signatures of TME established using the ESTIMATE algorithm, we initially integrated gene expression and DNA methylation information to develop a methylation-regulated TME prognostic signature. Additionally, our study is more comprehensive and in-depth than previous studies, which lacked research on immune therapy response and chemotherapy drug sensitivity [40,41,42]. In this study, TIDE scores were utilized to assess patient response to immune therapy. The low-risk group showed lower TIDE scores, indicating reduced immune evasion and greater immune therapy benefits. Furthermore, when combining immune scores, stroma scores, and TMB to predict patient immune therapy response, it was found that the prediction efficacy was significantly improved by the combined stroma scores. These findings further validate the efficacy of risk scores as prognostic markers for predicting patient immune responses. Additionally, in terms of drug prognosis, it was found that Imatinib, Paclitaxel, and Vinblastine were more suitable for high-risk patients, while Gemcitabine, 5-Fluorouracil, Sorafenib, pazopanib, and Methotrexate were more suitable for low-risk patients. These studies provide more evidence for precision medicine in ccRCC.

Activated CD8^+^ T cells, NK cells, and M1 macrophages can recognize and eliminate tumor cells [43,44,45], thereby directly inhibiting tumor growth. Conversely, the increase in Tregs, M2 macrophages, and MDSCs reshapes the TME, reducing the activity of other immune cells through various mechanisms, thereby promoting the immune evasion of tumors [46,47,48]. Our research findings indicate that the proportion and function of Tregs and MDSCs are higher in the high-risk group, suggesting the presence of an immunosuppressive TME, inhibiting the activation of CD8^+^ T cells, reducing the antigen presentation of dendritic cells, and thereby promoting immune evasion of tumors.

Tumors exhibiting elevated TMB typically harbor a greater quantity of mutated antigens, rendering them more readily identifiable and vulnerable to immune-mediated targeting. Hence, in cases of tumors exhibiting elevated TMB, the potential efficacy of immunotherapy may be heightened [49,50]. In this study, TMB in the low-risk group was lower, which contradicts the conclusion that the low-risk group is more suitable for immunotherapy. The reason for the better efficacy of immunotherapy in the low-risk group may not be solely attributed to TMB, as suggested by our speculation.

Pazopanib is a multi-target tyrosine kinase inhibitor used as a first-line treatment for ccRCC. In this study, it is suitable for low-risk patients. However, high-risk patients show decreased sensitivity to pazopanib. Recent studies have elucidated the presence of a CD133+/CD24+ cell subpopulation in ccRCC, which promotes angiogenesis within the TME and contributes to resistance against multitarget tyrosine kinase inhibitors, leading to tumor progression and poor prognosis. The relationship between high-risk patients and the CD133+/CD24+ cell subpopulation requires further investigation [36,51]. Additionally, expressions of *AJAP1* and *SLC6A19* are negatively correlated with the IC50 of pazopanib, while expressions of *MYH14* and *HOXB9* were positively correlated with the IC50 of pazopanib. These results suggest a close relationship between pazopanib and the prognostic signature related to methylation-regulated TME, as well as the four TME-MDGs used to construct the prognostic signature. Molecular docking analysis revealed that pazopanib has good binding affinity with the four TME-MDGs, all of which are less than −5 kcal/mol, suggesting that these four TME-MDGs may be potential targets of pazopanib. Interestingly, Serena Contarelli et al. [52] summarized that *HOXB9* has been experimentally validated to be associated with anti-angiogenesis and may be a potential target for anti-angiogenic drug therapy. This finding is highly consistent with our study, as we predict a strong binding affinity between HOXB9 and pazopanib. This may suggest that HOXB9 could be a potential undiscovered binding target for pazopanib. Recent studies have shown that MUC1 is phosphorylated by receptor tyrosine kinases and interacts with HIF-1a through the PI3K/Akt/mTOR pathway, regulating angiogenesis [53]. In this study, the expression of four TME-MDGs is regulated by DNA methylation and associated with the sensitivity of multiple multitarget tyrosine kinase inhibitors such as Sorafenib, pazopanib, Cabozantinib, Tivozanib, Axitinib, and Sunitinib. Additionally, in the molecular docking results, the four TME-MDGs showed a strong binding affinity with pazopanib. Multitarget tyrosine kinase inhibitors can directly inhibit MUC1 phosphorylation mediated by receptor tyrosine kinases, thereby regulating angiogenesis. This provides further evidence for the epigenetic regulation of angiogenesis by MUC1 in ccRCC.

A few limitations exist in this study. Firstly, this study is retrospective in nature, further validation of the prognostic signature’s accuracy requires large-scale prospective studies for confirmation. Secondly, our research data are primarily based on the TCGA, GEO, and ArrayExpress databases. Although preliminary validation was obtained in clinical samples, the sample size needs to be expanded, and more longitudinal prognostic data should be collected and included to thoroughly investigate the performance of the prognostic model in predicting long-term patient outcomes.

## 4. Materials and Methods

### 4.1. Clinical Sample Collection

This study was approved by the Biomedical Ethics Committee of Chongqing Medical University (approval number: 2024012), and all samples were obtained from the Department of Urology, the First Affiliated Hospital of Chongqing Medical University. All participants provided informed consent prior to surgery. We collected 15 cases of surgically resected ccRCC samples and matched adjacent normal tissue samples. Postoperative pathological reports confirmed all samples as ccRCC. The tissue samples were then cut into small particles and stored in RNAlater solution (Qiagen, Hilden, Germany) after washing with PBS (Sigma-Aldrich, St. Louis, MO, USA). The tissue samples were initially stored at −4 °C for one day and then stored at −80 °C. 

### 4.2. Data Collection

The training dataset was sourced from the TCGA website (https://www.cancer.gov/, accessed on 16 August 2023), encompassing transcriptomic data and clinical information for 72 normal kidney tissues and 525 ccRCC samples. Somatic mutation data were also collected from the TCGA website. DNA methylation data of 318 ccRCC and 160 normal kidney tissues were downloaded from the TCGA website. Microarray data and clinical information for the validation cohort were obtained from the GEO database (https://www.ncbi.nlm.nih.gov/geo/, accessed on 5 August 2023). The GSE167573 cohort comprised 55 ccRCC samples. The GSE53757 cohort comprised 72 ccRCC samples and 72 normal kidney tissue. The GSE206049 cohort comprised 30 normal kidney tissue samples and 138 ccRCC samples. The microarray data and clinical information of another validation cohort were obtained from the ArrayExpress dataset (https://www.ebi.ac.uk/arrayexpress, accessed on 27 September 2023). The E-MTAB-1980 cohort included 101 ccRCC samples. Protein data were obtained from the CPTAC database (https://proteomics.cancer.gov/, accessed on 7 June 2024). The PDC000127 cohort includes 110 ccRCC samples and 84 normal kidney tissue. Immunohistochemical and immunofluorescence images of two prognostically relevant genes were obtained from the HPA database (https://www.proteinatlas.org/, accessed on 27 January 2024).

### 4.3. Analysis of TME Based on ESTIMATE Algorithm

ESTIMATE-analyzed transcriptomic data enable us to estimate immune cells, stromal cells, and tumor purity in ccRCC and compare their differences across different clinical stages. The ccRCC samples were stratified into two groups based on the median values of immune scores, stromal scores, and ESTIMATE scores. Then we conducted a survival analysis and plotted KM survival curves.

### 4.4. Differential Expression and DNA Methylation Analysis

The “DESeq2” R package (version 1.30) was applied to analyze RNA-seq data, identifying DEGs between normal kidney tissues and ccRCC. Adjusted *p* < 0.05 and |log_2_FC| > 0.5 were taken as cut-off values to define DEGs.

The “CHAMP” R package (version 2.31.0) was used to evaluate DNA methylation data and investigate DMCs between normal kidney tissues and ccRCC. Probe information was annotated, mapping it to genes and identifying these genes as DMGs with a threshold of |Δβ| > 0.2 and adjusted *p* < 0.05.

Differential analysis between these two groups divided based on the median immune scores for TME-related genes was conducted using the “DESeq2” R package, with adjusted *p* < 0.05 and |log_2_FC| > 0.5 as thresholds.

### 4.5. Construction and Validation of Prognostic Signature Associated with Methylation Driven TME

Univariate Cox regression analysis initially screens for prognostic genes associated with methylation-regulated TME. After that, redundant genes were removed, and data dimensions were decreased using LASSO regression analysis. To simplify the prognostic signature, lambda.1se was selected, and, finally, a multivariate Cox regression analysis was conducted to construct the prognostic signature. The risk scores for ccRCC samples were calculated using the following equation: Risk scores = (−0.159 × expression of *MYH14*) + (0.178 × expression of *HOXB9*) + (−0.086 × expression of *SLC6A19*) + (−0.281 × expression of *AJAP1*).

KM survival curve analysis was then performed between the two groups divided based on the median risk scores. The “RMS” R package (version 6.7.1) and the “survival” R package (version 3.5.7) were employed to craft a predictive nomogram for survival rates. Additionally, the “time ROC” R package (version 0.4) was employed to generate ROC curves for evaluating the signature. To evaluate how well the actual and expected survival rates matched, calibration curves were utilized.

### 4.6. Infiltration of Immune Cells in ccRCC

Both the ssGSEA algorithm and the CIBERSORT algorithm were employed to assess the degree of immune cell infiltration in ccRCC. Immune cell enrichment scores are determined using the ssGSEA algorithm utilizing 29 immune-related features. Analysis was implemented using the “GSVA” R package (version 1.48), utilizing immune gene sets derived from the previous literature. The CIBERSORT algorithm employs linear regression analysis on gene expression data, using a reference immune cell gene set, to infer the relative proportions of 22 leukocyte subtypes in the samples. The analysis was performed using a validated leukocyte gene signature matrix (LM22) for the deconvolution [54].

### 4.7. Mutation Analysis

Employing the “mafTools” R package (version 2.16.0), mutation signature analysis was performed on the downloaded mutation data, and waterfall plots were generated to illustrate the mutation landscape of ccRCC patients. Furthermore, the following equation was applied to determine each sample’s TMB value: sample TMB = number of gene mutations/size of the exon region.

### 4.8. Drug Sensitivity and Immune Therapy Analysis

The ‘OncoPredict’ R package (version 0.2) estimates the IC50 of 198 anticancer drugs by evaluating the gene expression of tumor samples [55]. Additionally, a non-paired *t*-test was employed to scrutinize drug sensitivity, considering *p* < 0.05 as the significance threshold. Patients’ response to ICB therapy is assessed through the TIDE scores (http://tide.dfci.harvard.edu/, accessed on 27 January 2024).

### 4.9. Molecular Docking

The binding sites between pazopanib and four TME-MDGs were investigated using molecular docking analysis. The three-dimensional structure of pazopanib was obtained from the PubChem database (https://pubchem.ncbi, accessed on 27 January 2024) and converted using ChemBio3D (version 20.0). The protein structure data for SLCA19 and MYH14 were downloaded from the PDB database (https://www.rcsb.org/, accessed on 27 January 2024), while the data for HOXB9 and AJAP1 were obtained from the AlphaFold database (https://alphafold.com/, accessed on 27 January 2024). AutoDock software (version 4.2.6) was used to dock the core targets of the four TME-MDGs with pazopanib, and the resulting docking results were formatted using OpenBabel software (version 2.4.1). The PyMOL software (version 2.5.0) was used for visualization.

### 4.10. Genomic Alterations and CNV Analysis

The mutation locations, mutation quantities, and mutation types of the four TME-MDGs were analyzed using the cBioPortal database, and the relationship between different types of CNV and gene expression was evaluated. The types of CNV and their corresponding proportions for the four TME-MDGs in ccRCC were assessed using the GSVA website.

### 4.11. Protein Expression and Pathway Alteration Analysis

Using the ULCAN database, changes in protein levels of MYH14 and SLC6A19 were analyzed in mTOR pathway-altered, WNT pathway-altered, p53/Rb pathway-altered, RTK pathway-altered, MYC/MYCN-altered, Chromatin Modifier-altered, NRF2 pathway-altered, SWI-SNF complex-altered, and HIPPO pathway-altered conditions.

### 4.12. RNA Extraction and qRT-PCR

Total RNA was extracted using an RNaiso PLUS kit (Takara, Japan). Subsequently, 1.2 μg of RNA was reverse transcribed into cDNA using the Eastep@ RT Master Mix Kit (Promega, Beijing, China). The qRT-PCR system consists of 2× Universal SYBR Green Fast qPCR Mix (5 Μl; Qiagen, Hilden, Germany), DEPC-treated water (3.2 μL; Qiagen, Hilden, Germany), primer pairs (0.3 μL; Qiagen, Hilden, Germany), and cDNA (1.2 μL). The reaction conditions include pre-denaturation (95 °C, 3 min, 1 cycle), thermal cycling (95 °C, 10 s and 60 °C, 30 s, 40 cycles), and dissociation. *GAPDH* is used as an internal reference control, and mRNA relative expression is determined using the 2^−ΔΔCT^ method. The primer sequences are as shown in Table 2.

## 5. Conclusions

To summarize, our study provides insight into the relationship between prognosis and TME-MDGs in ccRCC. A reliable signature has been constructed and validated in two independent cohorts. Furthermore, relationships between the prognosis-related signature and TME, immune checkpoint expression, chemotherapy drug sensitivity, somatic mutations, and pathological stage have been explored. This provides personalized prognosis prediction for ccRCC patients. Moreover, there is a correlation between the IC50 of pazopanib and the expression of the four TME-MDGs. Molecular docking analysis reveals that pazopanib has a strong binding affinity with these four TME-MDGs, indicating that they may serve as therapeutic targets.

## Figures and Tables

**Table 1:** The predicted binding affinity of the proteins encoded by the four TME-MDGs to pazopanib.

Gene Symbol	Drug	Predictive Binding Energy (Kcal/mol)
*AJAP1*	pazopanib	−5.74
*MYH14*	pazopanib	−6.90
*SLC6A19*	pazopanib	−7.36
*HOXB9*	pazopanib	−5.03

**Table 2:** The primer pairs for qRT-PCR.

Gene	Forward Primers (5′−3′)	Reverse Primers (5′−3′)
*AJAP1*	CACGGCCTATAACGAGACCC	5′-TTCTCAGACACGAAGGCCAC
*MYH14*	GATTCAGCCACGAGGAAATCA’	5′-CGGTGTTCCGTTCTCTCTTCAA
*SLC6A19*	CATGGTGGGACTGTATTACAACA	CTCGTCCACATACCCTGTCTG
*HOXB9*	TCCAGCGTCTGGTATTTGGT	GAAGCGAGGACAAAGAGAGG’
*GAPDH*	GGTGTGAACCATGAGAAGTATGA	GAGTCCTTCCACGATACCAAAG

## Data Availability

This work relies on secondary analysis of publicly available databases. All data sources and acquisition times are mentioned in the Materials section of the article, and the data generated during the analysis process can be obtained by contacting the authors of the article.

## Supplementary Materials

The following supporting information can be downloaded at: https://www.mdpi.com/article/10.3390/ijms25126792/s1.
