# Supplementary material for: Identification and Validation of Tumor Microenvironment-Associated Signature in Clear-Cell Renal Cell Carcinoma through Integration of DNA Methylation and Gene Expression
Source: Int J Mol Sci. 2024 Jun 20;25(12):6792. doi: 10.3390/ijms25126792 (PMC11203551; doi:10.3390/ijms25126792)
Supplement: Supplementary file 1 [file ijms-25-06792-s001.zip › Revised supplementary figure.pdf]

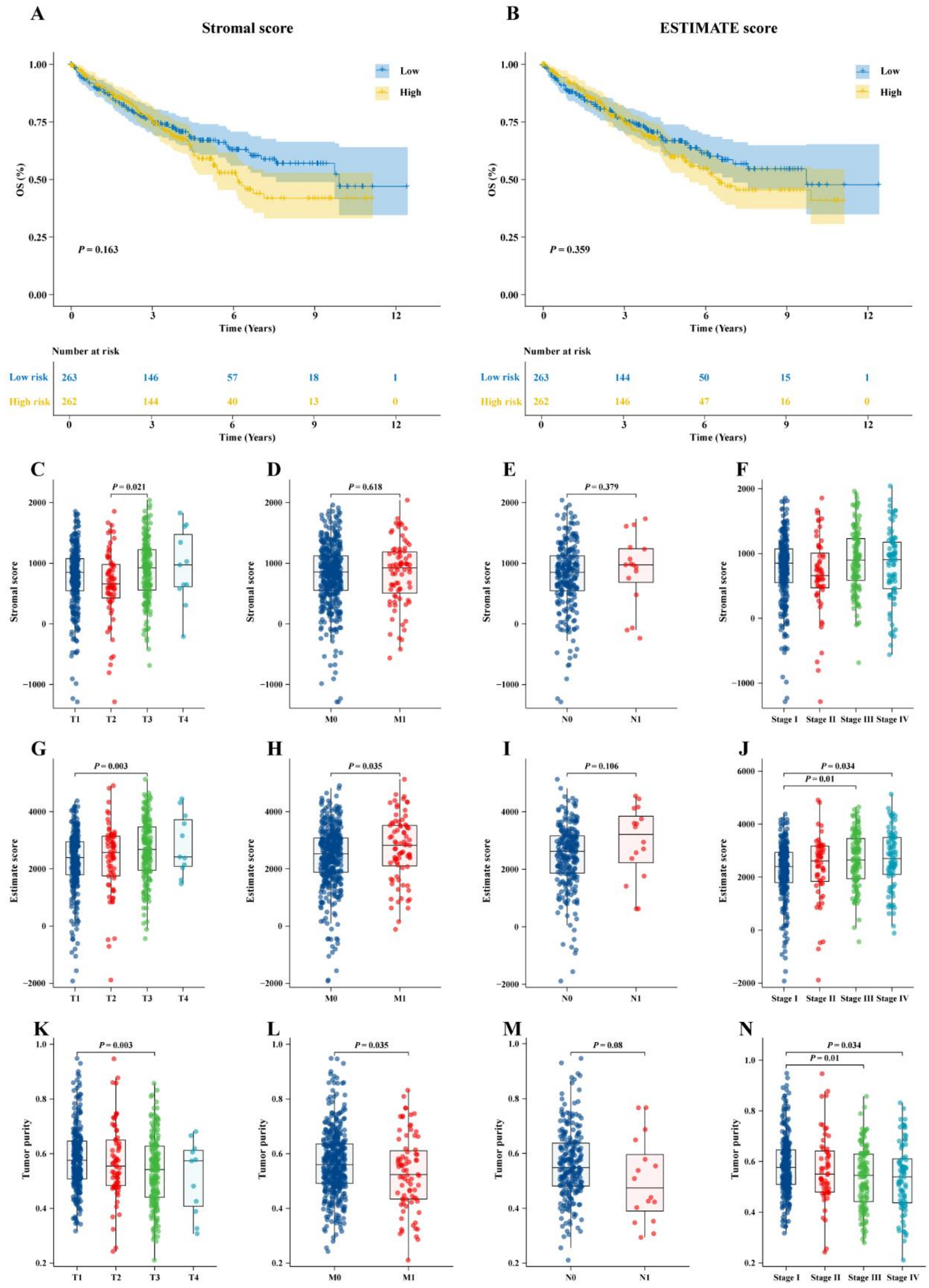

Supplementary Figure S1. TME in the TCGA-KIRC cohort. KM survival curves demonstrate the independent correlation of stromal score and ESTIMATE score with OS. (A-B) Box plots illustrate the distribution of stromal score, (C-F) ESTIMATE score, (G-J) and tumor purity (K-N) in TMN stage and pathological stage.

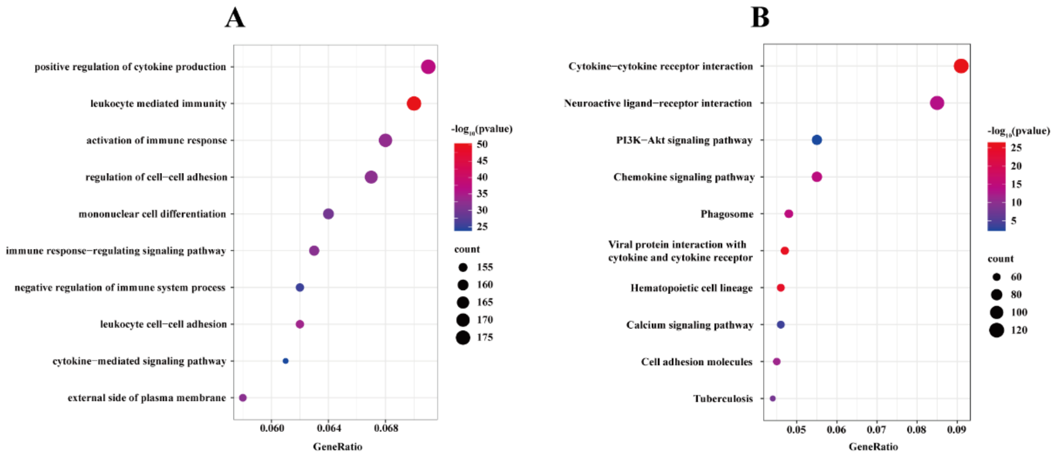

Supplementary Figure S2. Enrichment analysis of TME-related genes. Bubble plots illustrate the GO enrichment results (A) and KEGG enrichment results (B) in TME-related genes.

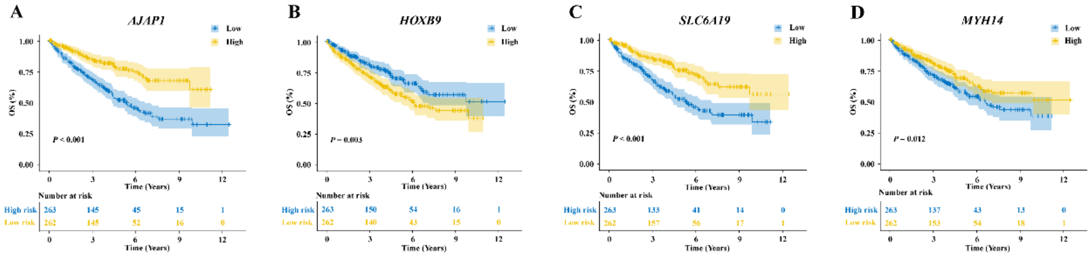

Supplementary Figure S3. The correlations between genes in prognostic signature and OS. KM survival curves demonstrate the independent correlation of (A) *AJAP1*, (B) *HOXB9*, (C) *SLC6A19*, and (D) *MYH14* with OS.

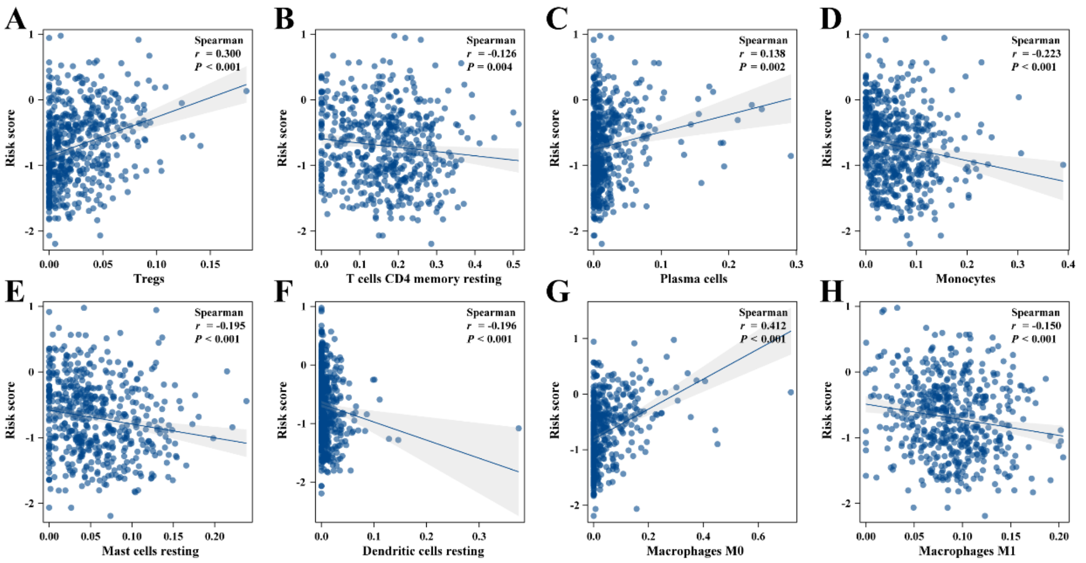

Supplementary Figure S4. The correlation analyses between immune cells in CIBERSORT and risk score. Correlation analysis between the scores of (A) Tregs, (B) T cells $CD4^+$ memory resting, (C) Plasma cells, (D) Monocytes, (E) Mast cells resting, (F) Dendritic cells resting, (G) Macrophage M0, and (H) Macrophage M1 and the risk score.

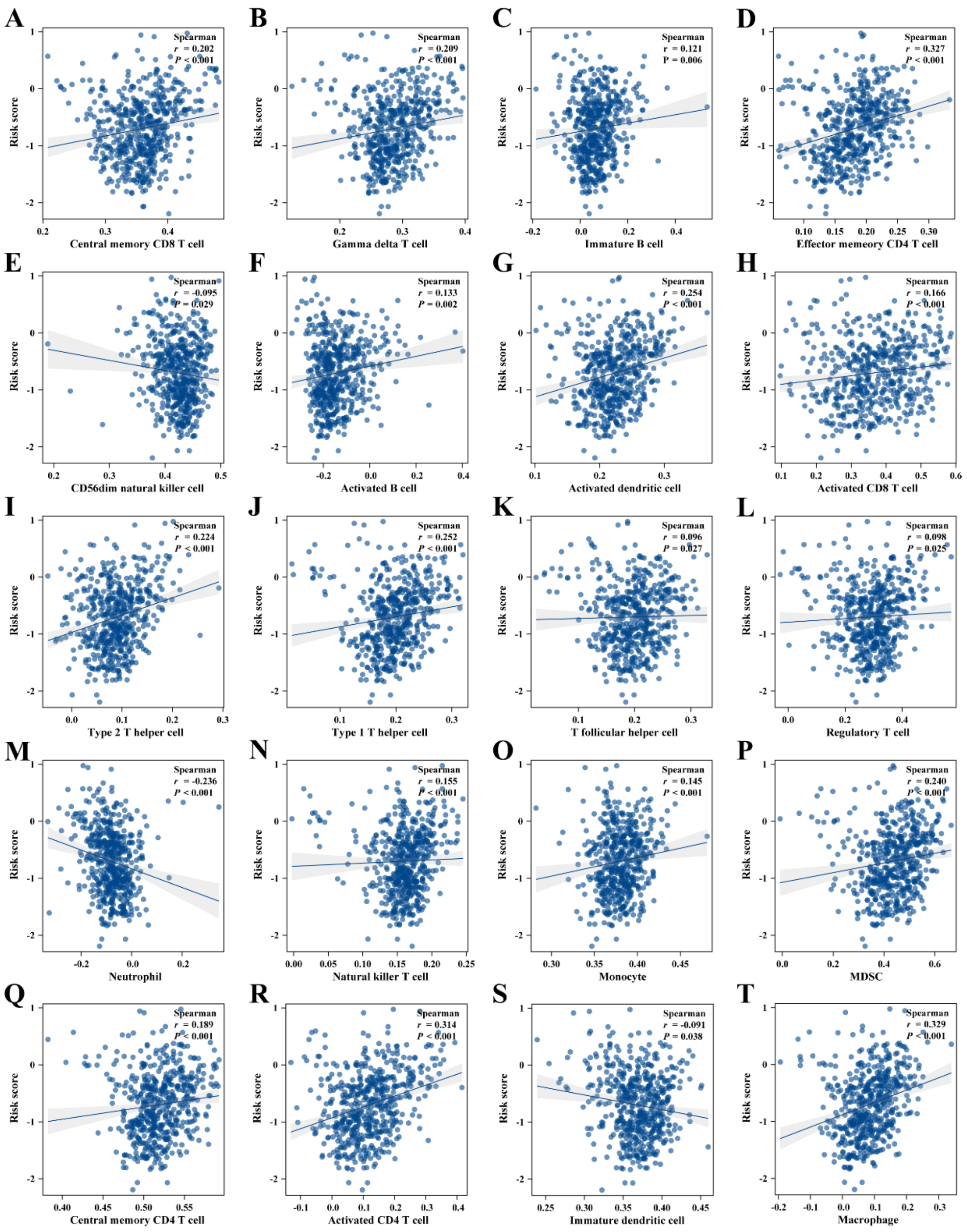

Supplementary Figure S5. The correlation analysis between immune cells in ssGSEA and risk score. Correlation analysis between the scores of (A) Central memory $CD8^+$ T cell, (B) Gamma delta T cell, (C) Immature B cell, (D) Effector memory $CD4^+$ T cell, (E) CD56dim natural killer cell, (F) Activated B cell, (G) Activated dendritic cell, (H) Activated $CD8^+$ T cell, (I) Type 2 T helper cell, (J) Type 1 T helper cell, (K) T follicular

helper cell, (L) Regulatory T cell, (M) Neutrophil, (N) Natural killer T cell, (O) Monocyte, (P) MDSC, (Q) Central memory CD4^{+} T cell, (R) Activated CD4^{+} T cell, (S) Immature dendritic cell, (T) Macrophage, and the risk score.

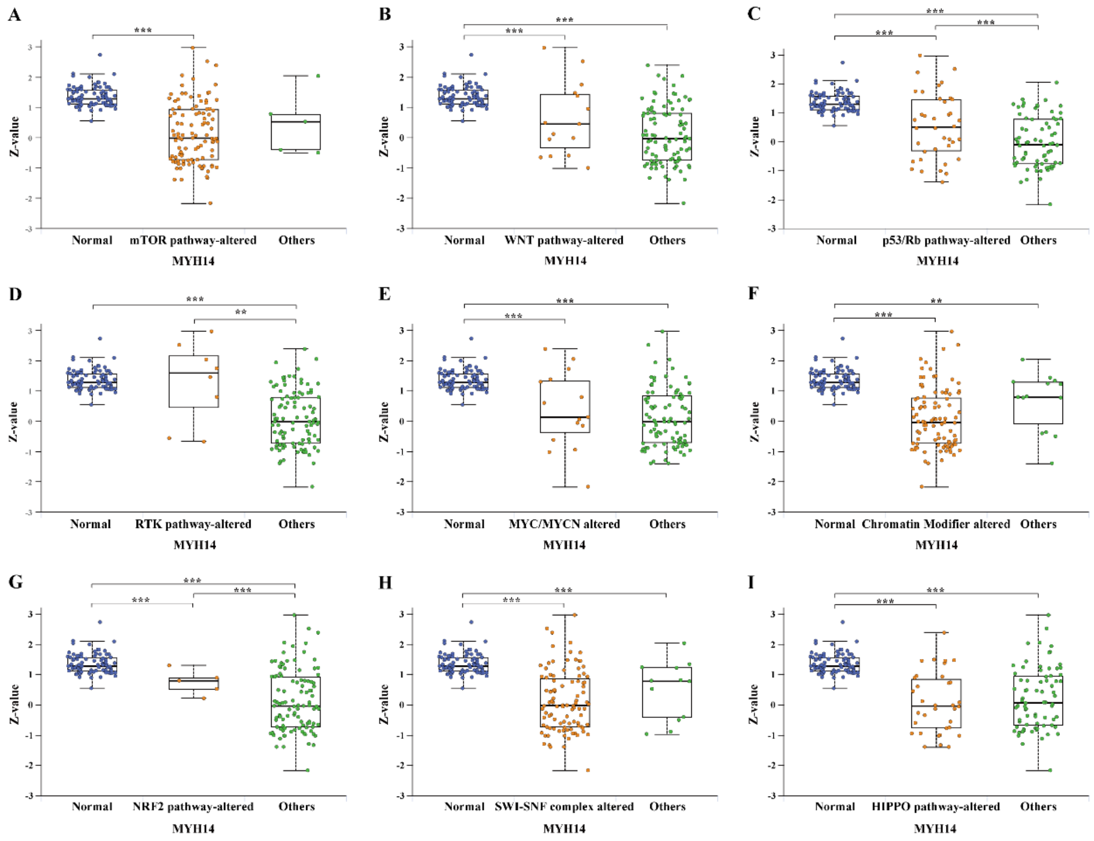

Supplementary Figure S6. Evaluate the differences in MYH14 protein expression across nine pathway alterations. Assess the changes in MYH14 protein expression in (A) mTOR pathway-altered, (B) WNT pathway-altered, (C) p53/Rb pathway-altered, (D) RTK pathway-altered, (E) MYC/MYCN altered, (F) Chromatin Modifier altered, (G) NRF2 pathway-altered, (H) SWI-SNF complex altered, and (I) HIPPO pathway-altered conditions; ** $P < 0.01$, *** $P < 0.001$.

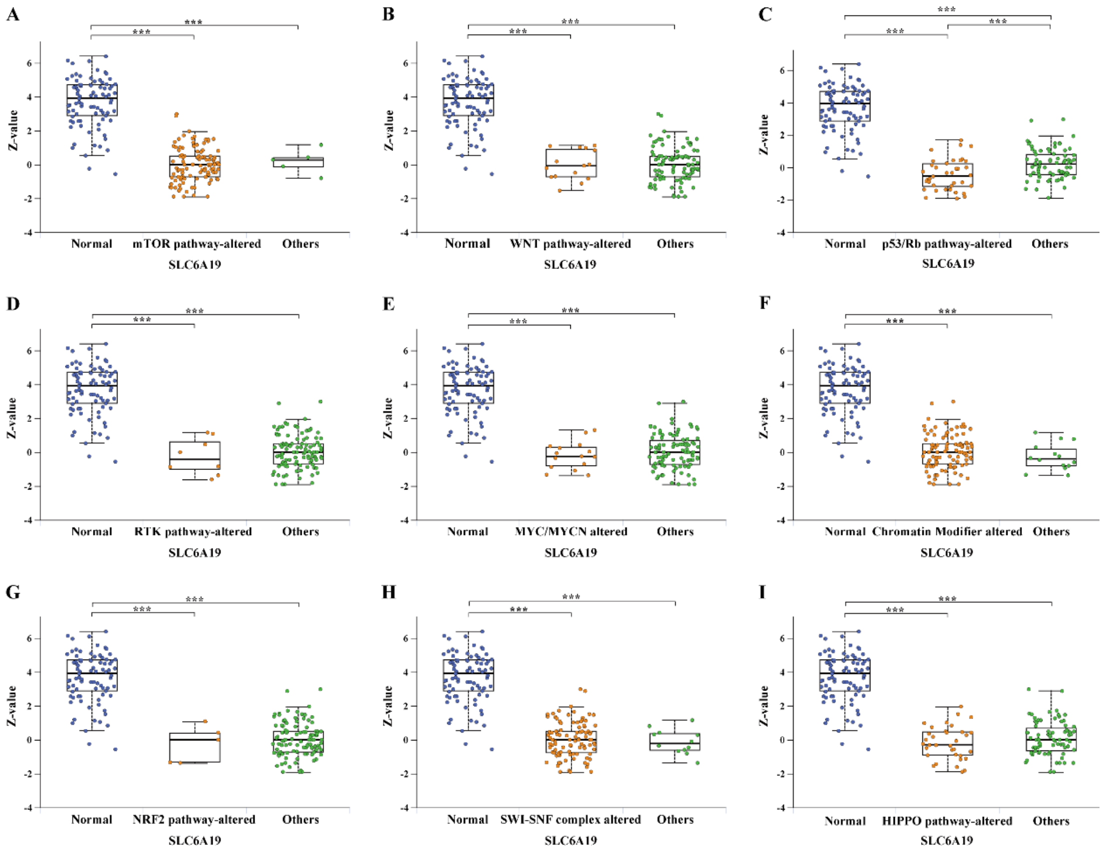

Supplementary Figure S7. Evaluate the differences in SLC6A19 protein expression across nine pathway alterations. Assess the changes in SLC6A19 protein expression in (A) mTOR pathway-altered, (B) WNT pathway-altered, (C) p53/Rb pathway-altered, (D) RTK pathway-altered, (E) MYC/MYCN altered, (F) Chromatin Modifier altered, (G) NRF2 pathway-altered, (H) SWI-SNF complex altered, and (I) HIPPO pathway-altered conditions; *** $P < 0.001$.
